# RelQ Mediates the Expression of β-Lactam Resistance in Methicillin-Resistant *Staphylococcus aureus*

**DOI:** 10.3389/fmicb.2019.00339

**Published:** 2019-03-11

**Authors:** Ajita Bhawini, Parul Pandey, Ashutosh Prakash Dubey, Aafreen Zehra, Gopal Nath, Mukti Nath Mishra

**Affiliations:** ^1^Department of Microbiology, Institute of Medical Sciences, Banaras Hindu University, Varanasi, India; ^2^School of Biotechnology, Faculty of Science, Banaras Hindu University, Varanasi, India; ^3^Biotechnology Division, CSIR-Central Institute of Medicinal and Aromatic Plants, Lucknow, India

**Keywords:** RelP, RelQ, MRSA, MecA, Stringent Response

## Abstract

An induced stringent response, which is established by an increased level of (p)ppGpp, is required for the expression of β-lactam resistance in methicillin-resistant *Staphylococcus aureus* (MRSA). However, it is not clear whether RSH (enzyme mediating stringent response to amino acid starvation) or small alarmone synthetases (SASs) are involved in the maintenance of (p)ppGpp level in response to β-lactams. Since the *S. aureus* genome encodes two active SASs (RelP and RelQ), their contribution to the expression of β-lactam resistance in MRSA was investigated. It was determined that *relQ* deletion renders community-associated MRSA (CA-MRSA) sensitive to β-lactams by negatively affecting the expression of *mecA*, and induction of (p)ppGpp synthesis by mupirocin bypasses the requirement of *relQ* for the expression of high-level β-lactam resistance. Surprisingly, *relP* deletion increased the level of β-lactam resistance. Such contradictory observations could be attributed to the fact that *relQ* promoter is ~5-fold stronger than the *relP* and is induced by oxacillin as well as deletion of either of the SASs, while *relP* promoter responds only to oxacillin. The stronger promoter activity of *relQ*, coupled with the inducibility of the *relQ* promoter in response to the lack of *relP*, results in efficient expression of *relQ* in the *relP*-deleted background. This positively affects *mecA* expression and renders the Δ*relP* strain highly resistant. These findings indicate an important role for RelQ in the expression of high-level β-lactam resistance in MRSA.

## Introduction

*Staphylococcus aureus* is a Gram-positive cocci often found on human and animal skins and mucous membranes. It is commonly associated with opportunistic infections in hospitals and the community. Methicillin-resistant *S. aureus* (MRSA) is intrinsically resistant to most of the β-lactams due to the presence of the *mecA* gene encoding an altered penicillin-binding protein (PBP) known as PBP-2a (Llarrull et al., 2009). In methicillin-susceptible *S. aureus* (MSSA), the transpeptidase activity of PBPs is lost due to irreversible acylation of an active site serine by the β-lactam antibiotics (Williamson and Tomasz, 1985). However, the PBP-2a of MRSA is resistant to β-lactam acylation, and successfully catalyzes the DD-transpeptidation reaction, leading to a methicillin-resistant phenotype (Fuda et al., 2004). Although *mecA* is essential for methicillin resistance, it in itself is not sufficient, since the native PBP2 is also required for MRSA (Pinho et al., 2001) and the level of resistance expressed could be altered by varying temperature, pH, and salt concentration (Chambers and Hackbarth, 1987).

The stringent response (SR) is a highly conserved regulatory mechanism induced by amino acid starvation and various environmental stresses and is established by (p)ppGpp (nutritional alarmone) synthesis. This response adjusts the cell's biosynthetic machinery according to the availability of required precursors and energy. In *Escherichia coli*, (p)ppGpp is synthesized by two cytoplasmic enzymes, a (p)ppGpp synthetase (RelA), and a (p)ppGpp synthetase/hydrolase (SpoT) (Xiao et al., 1991). Both these enzymes are composed of N-terminal enzymatic (synthetase and/or hydrolase) and C-terminal regulatory domains (Mechold et al., 2002). *Staphylococcus aureus* harbors a Rel/SpoT homolog (RSH) and two additional small *a*larmone *s*ynthetases (SASs) encoded by *relP* and *relQ* (Eymann et al., 2002; Nanamiya et al., 2008; Geiger et al., 2010, 2014). The RSH has N-terminal enzymatic (synthetase and hydrolase) and C-terminal regulatory domains and performs both (p)ppGpp synthesis and degradation activities. Each SAS (RelP or RelQ) has only a synthetase domain; both the hydrolase and the C-terminal regulatory domains are missing.

The role and regulation of RelP and RelQ has been studied in *Bacillus subtilis* (Nanamiya et al., 2008), *Streptococcus mutans* (Lemos et al., 2007), and *Enterococcus faecalis* (Abranches et al., 2009). These studies showed that *relP* and *relQ* encode active (p)ppGpp synthatases, which are dispensable in optimal growth conditions. In MSSA, it has been shown that *relP* and *relQ* encode active (p)ppGpp synthatases, which are induced in response to cell wall-active antibiotics to mitigate such conditions (Geiger et al., 2014). Recently, ethanol-mediated induction of *relP* in MRSA was reported (Pando et al., 2017), but involvement of these SASs in the expression of β-lactam resistance in MRSA is still unclear. Here, we explore the involvement of RelP and RelQ in the expression of β-lactam resistance in a community-associated MRSA (CA-MRSA) strain by deleting *relP* and *relQ* and characterizing the mutants. This study reveals the importance of RelQ for full expression of β-lactam resistance in MRSA and confirms that *relQ* deletion negatively affects the *mecA* expression. The transcript and promoter activity analyses showed that the apparent opposite effect of *relP* (positive) and *relQ* (negative) deletions on the level of β-lactam resistance is a consequence of enhanced *relQ* induction in the *relP*-deleted background.

## Materials and Methods

### Bacterial Strains, Plasmids, and Growth Conditions

The bacterial strains and plasmids used in this study are described in Table 1. Mueller-Hinton (MH) medium was used for drug susceptibility tests. Tryptic Soya Broth (TSB) and Tryptic Soya Agar (TSA) were used for mutagenesis work. Plasmids were propagated and maintained in *E. coli* DH5α (Taylor et al., 1993) and *S. aureus* RN4220 (Kreiswirth et al., 1983) using 100 μg/ml ampicillin (Amp) and 10 μg/ml chloramphenicol (Cm), respectively. The nucleotide sequence of the primers used in this study are listed in Table 2.

**Table 1:** Bacterial strains and plasmids used in this study.

**Strains or plasmids**	**Relevant properties**	**References or sources**
***E. coli*** **STRAINS**		
DH5α	Δ*lacU*169 *hsdR*17 *recA1 endA*1 *gyrA*96 *thiL relA1*	Taylor et al., 1993
***S. aureus*** **STRAINS**		
RN4220	Restriction-deficient *S. aureus* strain	Kreiswirth et al., 1983
JE2	Wild-type; developed from a CA-MRSA, USA300-FPR3757	Fey et al., 2012
*ΔrelP*	*relP* deletion mutant of JE2	Pando et al., 2017
*ΔrelQ*	*relQ* deletion mutant of JE2	This work
*ΔrelPQ*	*relPQ* deletion mutant of JE2	This work
**PLASMIDS**		
pLI50	*E. coli-S. aureus* shuttle plasmid; Amp^R^ (*E. coli*); Cm^R^ (*S. aureus*)	Lee et al., 1991
pKOR1	ATc-inducible suicide mutagenesis vector	Bae and Schneewind, 2006
pALC2073	*E. coli-S. aureus* shuttle plasmid with *xyl/tetO* promoter;	Bateman et al., 2001
pKOR1-*ΔrelQ*	*relQ* deletion plasmid	This work
pMN12	*relP* ORF with its native promoter cloned in KpnI/XbaI site of pLI50	This work
pMN13	*relQ* ORF with its native promoter cloned in KpnI/SalI site of pLI50	This work
pMN14	*relP* promoter fused with *relQ* ORF and cloned in KpnI/SalI site of pLI50	This work
pMN15	*relQ* promoter fused with *relP* ORF and cloned in KpnI/XbaI site of pLI50	This work
pMN18	*E. coli lacZ* engineered ORF cloned in XbaI/HindIII sites of pLI50 with *Bacillus subtilis spoVG* ribosomal binding site.	This work
pMN19	*relP* promoter region cloned in KpnI/XbaI sites of pMN18	This work
pMN20	SAUSA300_0906 promoter region cloned in KpnI/XbaI sites of pMN18	This work
pMN21	*relQ* upstream region (including SAUSA300_0906 promoter and ORF) cloned in KpnI/XbaI sites of pMN18	This work
pMN25	*relQ* ORF cloned in EcoRI sites of pALC2073	This work
pMN26	*mecA* ORF cloned in EcoRI sites of pALC2073	This work

*Amp^R^, ampicillin resistance; Cm^R^, chloramphenicol resistance*.

**Table 2:** Primers used in this study (additional nucleotides not specific for *S. aureus* are shown in bold; restriction sites are underlined; and nucleotides used to produce regions for overlap PCR are italicized).

**Primer**	**Sequence (5**′** to 3**′**)**
attB1-relQ-us:F	**GGGGACAAGTTTGTACAAAAAAGCAGGCT**CAATGATGTCATATGGTGTTGTTG
relQ-us:R:BamHI	**CG****GGATCC**CCATTGATTCATAGTGCTTCACC
relQ-ds:F:BamHI	**CG****GGATCC**GATTAACGAGGTGTTATAAATCATG
attB2-relQ-ds:R	**GGGGACCACTTTGTACAAGAAAGCTGGGT**GTTGTATTACGATCTAGACGCGTAAC
relP-up:F	GATTGGTATCGAGCGTTATCG
relP:R	CACACCTACTAAACATCTACTC
relQ-up:F	GTCGTTAATGCACCAAGTATTG
relQ:R	AAGGCATTAGACTTGGAGTCAC
rpoB:F	GTGACGCTACTTATGCTGCAC
rpoB:R	CGAACGTACCTGTATCAGTC
pbp2:RT:F	AGCGTATGGACCTGCCATTG
pbp2:RT:R	GTACCGTGACTCTTCGTATC
mecA:RT: F	CACCTTCATATGACGTCTATC
mecA:RT:R	GAACCTGGTGAAGTTGTAATC
PrelP:F:KpnI	**CGG****GGTACC**AGAGAACCGCTTATGGATGGTCCAC
relP:R:SalI	**ACGC****GTCGAC**CACACCTACTAAACATCTACTC
P0906:F:KpnI	**CGG****GGTACC**GCAATTTATTATAGATTGATGCAGTTATC
relQ:R:SalI	**ACGC****GTCGAC**AGGCATTAGACTTGGAGTCAC
PrelP-(Q):R	*CTGATCCCATTGATT*CATTTTTATACTAACCTC
relQ-ORF(P):F	*GAGGTTAGTATAAAA*ATGAATCAATGGGATCAG
P0906-(P):R	*GTTTTCGATCTACATA*CATGCTTAATCCTCCTCTTATTC
relP-ORF(Q):F	*GAATAAGAGGAGGATTAAGC*ATGTATGTAGATCGAAAAC
lacZ:SVF:XbaI	**TGC****TCTAGA****GGGAAAAGGTGGTGAACTACTGTGGAAGTTACTG**
lacZ:SVF	**AACTACTGTGGAAGTTACTGACGTAAGATTACGGGTCGACTGGGAAAACCCTGGCGTTAC**
lacZ:R:BglII	**GA****AGATCT****CTGCCCGGTTATTATTATTTTTGACACCAGACCAACTG**
TF:BglII	**GA****AGATCT****GCGATGGCTGTTTTGGCGGATGAGAG**
TR:HindIII	**CCC****AAGCTT****GTTTGTAGAAACGCAAAAAGGCCATC**
PrelP:R:XbaI	**GC****TCTAGA**CGATATATAATCATCTTTATTGTACC
P0906:R:XbaI	**GC****TCTAGA**TCCATAACATTTTAACACAATTCAATAATAC
PrelQ:R:XbaI	**GC****TCTAGA**CTTTATTCAATGTCGAATGTTTCTTC
relQ:F:EcoRI	**G****GAATTC**ATAAAGCGGGGTGAAGCACTATG
relQ:R:EcoRI	**G****GAATTC**TGATTTATAACACCTCGTTAATC
mecA:F:EcoRI	**G****GAATTC**GTCTTATATAAGGAGTATATTGATG
mecA:R:EcoRI	**G****GAATTC**TTATTCATCTATATCGTATTTTTTATTAC
relP-SP1:R	CCACATATCCATACCTATC
relP-SP2:R	CACACGTCGCTCCATATGATG
relP-SP3:R	GTGCTTATTTCCTTTAGTGCTGAC
relQ-SP1:R	CAATAATGACATGATACGAG
relQ-SP2:R	CTTAAACCAGCGATATCGTAC
relQ-SP3:R	CACCAACTTCATATTGTTTGCGCATG

### Mutant Construction

The markerless and in-frame *relQ* and *relPQ* deletion mutants were constructed using Gateway cloning technology-based ATc-inducible suicide mutagenesis vector pKOR1 (Bae and Schneewind, 2006), following the protocol that was used earlier for construction of Δ*relP* (Pando et al., 2017). Briefly, the upstream and downstream regions of *relQ* ORFs were amplified using attB1-relQ-us:F/relQ-us:R:BamHI and relQ-ds:F:BamHI/attB2-relQ-ds:R primer sets (Table 2), respectively, and ligated after BamHI digestion. Afterwards, the ligated fragments were inserted in pKOR1 using the BP reaction of Gateway Technology (Invitrogen) to construct *relQ* deletion plasmid designated as pKOR1-Δ*relQ*. The deletion plasmid was first mobilized into *S. aureus* RN4220, isolated from RN4220 and then transferred into JE2 (Fey et al., 2012) and Δ*relP* by electroporation. Deletion plasmid was integrated (single cross-over) into JE2 and Δ*relP* chromosome by growing the transformants at 43°C (non-permissive condition) in TSB supplemented with 7.5 μg/ml chloramphenicol. Integrated plasmid was excised (double cross-over) to replace the wild-type allele by growing at 30°C (permissive condition) in TSB supplemented with 10 μg/ml chloramphenicol, and plasmid-free cells were selected by growing at 30°C temperature on TSA plates supplemented with 1 μg/ml ATc.

### Antibiotic Susceptibility Tests

Drug sensitivity tests were performed by the Kirby–Bauer method as described earlier (Bauer et al., 1959), and the minimal inhibitory concentration (MIC) of oxacillin was determined using the agar double-dilution method as described by Wiegand et al. (2008). The MICs were determined using oxacillin concentrations ranging from 0.25 to 1,024 μg/ml in the absence and presence of mupirocin (0.03 μg/ml), and repeated >7 times to confirm the differences in antibiotic susceptibilities.

### Construction of *relP* and *relQ* Expression Plasmids

The *relP* and *relQ* genes were amplified with their native promoters using PrelP:F:KpnI/relP:R:SalI and P0906:F:KpnI/relQ:R:SalI primer sets (Table 2), respectively. The PCR products were cloned into the KpnI/SalI site of pLI50 (Lee et al., 1991) to construct pMN12 (pLI50-*relP*) and pMN13 (pLI50-*relQ*). To fuse *relQ* promoter with *relP* ORF, P0906:F:KpnI/P0906-(P):R and relP-ORF(Q):F/relP:R:SalI primer sets (Table 2) were used to amplify *relQ* promoter and *relP* ORF, respectively. P0906-(P):R and relP-ORF(Q):F primers are engineered to incorporate overlapping nucleotides in the amplified products to facilitate the overlap PCR. The overlap PCR was performed using PCR products as template with the P0906:F:KpnI/relP:R:SalI primer set. Similarly, *relP* promoter was fused with *relQ* ORF using PrelP:F:KpnI/PrelP-(Q):R and relQ-ORF(P):F/relQ:R:SalI primer sets (Table 2). Finally, fused PCR products, P_relQ_-*relP* and P_relP_-*relQ*, were cloned into KpnI/SalI sites of pLI50 to construct pMN14 (pLI50- P_relP_-*relQ*) and pMN15 (pLI50- P_relQ_-*relP*). Recombinant plasmids were first mobilized into *S. aureus* RN4220 by electroporation. Plasmids were isolated from RN4220 and then mobilized into parent and mutant strains by electroporation.

### Construction of *xyl/tetO* Promoter-Driven Expression Plasmids

The *relQ* and *mecA* ORFs were amplified with their native ribosomal binding sites (RBS) using relQ:F:EcoRI/relQ:R:EcoRI and mecA:F:EcoRI/mecA:R:EcoRI primer sets, respectively (Table 2). The PCR products were cloned into pALC2073 (Bateman et al., 2001) using the EcoRI site. Recombinant plasmids were confirmed by restriction digestion and sequencing, and designated as pMN25 (pALC2073-*relQ*) and pMN26 (pALC2073-*mecA*). These plasmids were mobilized in parent and mutant strains via *S. aureus* RN4220.

### RNA Extraction and Real-Time PCR

Cells were harvested from 1 ml of mid-log phase cultures (0.5–0.9 OD_600nm_) by centrifugation at 6,000 rpm for 5 min at 4°C, resuspended in 100 μl TE (30 mM Tris-Cl and 1 mM EDTA, pH 8.0), and lysed with 100 μg/ml lysostaphin (Sigma, USA) by incubating at 30°C for 5 min. Afterwards, TRIzol reagent (Invitrogen, Germany) was used to extract total RNA. RNA samples were treated with RNase-free DNase I (New England Biolabs) at a final concentration of 1 U/50 μl sample for 30 min at 37°C. RNA samples were used as template with *Taq* DNA polymerase to check DNA contaminations. For relative quantification, 2 μg total RNA was reverse transcribed using a cDNA Synthesis Kit (Fermentas, USA), and 1 μl of 10x diluted cDNA was used as template in 20 μl reaction volume. Real-time quantitative PCR (qPCR) was performed using SYBR Green Master mix (ROX; Fermentas) and the 7500 Real-Time PCR System (Applied Biosystems, CA, USA). An amplicon of *rpoB* was used as endogenous control for relative quantification by the $2^{-\text{Δ}\Delta {\text{C}}_{\text{T}}}$ method. Primer efficiencies were calculated by generating the standard curves for each primer pair. Efficiencies of the primer pairs used for RT-PCR were found to be 92–99%. ANOVA (analysis of variance) followed by Tukey's *post-hoc* test was performed using SPSS 17.0 software for data analysis, and *p*-values < 0.05 were considered as significantly different in relative expression level.

### Construction of *lacZ*:Reporter Vector and Promoter-*lacZ* Transcriptional Fusions

*Escherichia coli* genomic DNA was used as template with lacZ:SV:F/lacZ:R:BglII (Table 2) primers to amplify *lacZ* ORF. This amplified product was used as template with lacZ:SVF:XbaI/lacZ:R:BglII (Table 2) primers for the second round PCR amplification to add *B. subtilis spoVG* RBS and modify N-terminal of *lacZ* ORF as present in pMUTIN2 (Vagner et al., 1998). Primer lacZ:SVF:XbaI and lacZ:SV:F were designed with additional/engineered nucleotides to add *B. subtilis spoVG* ribosome-binding site and modified N-terminal of *lacZ* ORF as present in pMUTIN2. The nucleotide sequence of pMUTIN2 (GenBank accession No. AF072806) was followed for this manipulation. A 428 bp fragment encompassing t_1_ t_2_ terminators of the *E. coli rrnB* operon known to be active in *B. subtilis* (Peschke et al., 1985) was amplified using pMMB206 as template (Morales et al., 1991) with TF:BglII/TR:HindIII primers (Table 2). The *lacZ* ORF and the terminator were cloned into XbaI/HindIII restriction sites of pLI50 using three fragment ligation to construct pMN18. The upstream regions of *relP* (485 bp), SAUSA300_0906 (231 bp), and *relQ* (599 bp) were amplified using PrelP:F:KpnI/PrelP:R:XbaI, P0906:F:KpnI/0906:R:XbaI and P0906:F:KpnI/relQ-Pr:R:XbaI primers, respectively (Table 2), and cloned into KpnI/XbaI sites of pMN18 to construct *relP* (pMN19), SAUSA300_0906 (pMN20), and *relQ* (pMN21) promoter:*lacZ* fusions.

### β-Galactosidase Assay

*Staphylococcus aureus* strains harboring promoter:*lacZ* transcriptional fusions or the promoterless vector (pMN18) were grown in MHB up to mid-log phase (0.5–0.6 OD_600nm_). A culture of each strain was then divided into two parts. One part of each strain was supplemented with 4 μg/ml of oxacillin and incubated at 37°C for 60 min, while the other part was kept as control. The cell number in the treated and control cultures of different strains were equalized by adjusting OD_600nm_ = 0.5 using MHB with or without oxacillin. β-galactosidase assays were performed with 1 ml of adjusted culture as described earlier (Miller, 1972). ANOVA (analysis of variance) followed by Tukey's *post-hoc* test was performed using SPSS 17.0 software for data analysis, and *p*-values < 0.05 were considered as significantly different in β-galactosidase activities.

### 5′ RACE

The *relP* and *relQ* transcription start sites (TSS) were identified using a 3′/5′ RACE kit, 2nd Generation (Roche, Germany), following the manufacturer's protocol. Briefly, *relP* and *relQ* transcripts were reverse transcribed from total RNA into cDNA using relP- or relQ-SP1:R (Table 2). cDNAs were purified and 3′-poly(dA) tailed and then used as template in two PCRs designed to amplify the 5′ ends of *relP* and *relQ* using oligo(dT)-anchor/relP-SP2:R or oligo(dT)-anchor/relQ-SP2:R primers, respectively. First PCR products were separately used as template in second PCR using anchor/relP-SP3:R and anchor/relQ-SP3:R primer sets. PCR products were ligated into pGEM-T Easy (Promega, USA), and the clones were sequenced.

## Results

### Deletion of *relQ* Results in β-Lactam Sensitivity

The role of (p)ppGpp in increased/homogenous expression of β-lactam resistance has been demonstrated in *S. aureus* (Mwangi et al., 2013). However, contribution of RelP, RelQ, and RSH to maintain the (p)ppGpp level required for the expression of β-lactam resistance is not clear. To examine the contribution of RelP and RelQ to the expression of drug resistance, *relQ* was deleted in JE2 (a CA-MRSA, wild-type strain) and Δ*relP* following a previously described protocol (Pando et al., 2017). The in-frame deletion mutants, Δ*relQ* and Δ*relPQ*, were constructed by deleting 618 bp of *relQ* (636 bp) in JE2 and Δ*relP*, respectively, and confirmed by PCR amplification of the *relQ* locus, and sequencing of amplicons obtained from JE2 and mutants. As expected, the relP-up:F/relP:R (Table 2) primer set produced 1,209 bp fragment with JE2 or Δ*relQ* genomic DNA while a smaller amplicon of 532 bp was produced with that of Δ*relP* or Δ*relPQ* (Supplementary Figure 1). Similarly, the relQ-up:F/relQ:R (Table 2) primer set produced ~1,600 bp amplicon with JE2 or Δ*relP* genomic DNA, and a 998 bp amplicon with that of Δ*relQ* or Δ*relPQ* (Supplementary Figure 1). PCR amplification of expected amplicons and their sequencing confirmed the in-frame deletion of *relQ* in the mutant strains (data not shown). The *relQ* and *relPQ* deletion mutants were designated as Δ*relQ* and Δ*relPQ*, respectively.

**Figure 1:**
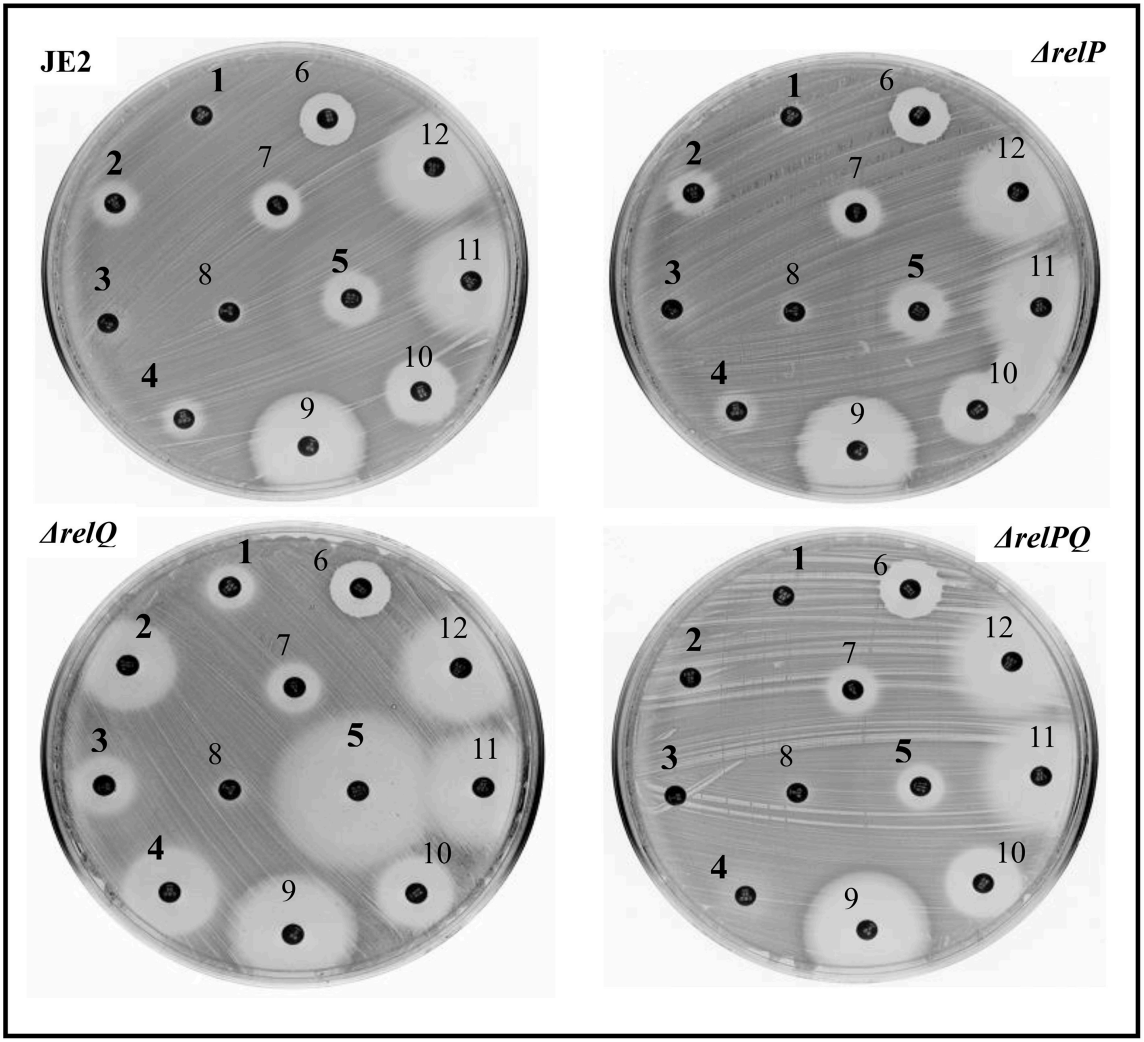
Disc-susceptibility test of JE2, Δ*relP*, Δ*relQ*, and Δ*relPQ* using Kirby-Bauer antibiotic testing. The numbers indicate different antibiotic disks; ceftazidime (**1**), cefepime (**2**), oxacillin (**3**), ceftriazone (**4**), tezobactam/piperacillin (**5**), vancomycin (6), streptomycin (7), ciprofloxacin (8), erythromycin (9), amikacin (10), linozelid (11), and spectinomycin (12). Numbers in bold indicate the disks for which differences in the sensitivity were observed.

Disc-susceptibility testing revealed that non-β-lactam antibiotic (vancomycin, streptomycin, ciprofloxacin, erythromycin, amikacin, linezolid, and spectinomycin) disks produced either no or an equal-size zone of inhibition with parent and mutant strains. However, β-lactam antibiotic (ceftazidime, cefepime, oxacillin, ceftriaxone, and tazobactam/piperacillin) disks produced a larger zone of inhibition with Δ*relQ* compared to the parent (Figure 1). Intriguingly, this test also revealed that the zone of inhibitions produced by β-lactams in the case of Δ*relP* and Δ*relPQ* were equal to that of the parent (JE2). Since Δ*relPQ* was less sensitive than Δ*relQ, relP* was again deleted in the Δ*relQ* strain, and the disc susceptibility test was repeated. This revealed that deletion of *relP* from the Δ*relQ* genome renders the strain Δ*relPQ* less sensitive compared to that of Δ*relQ* (data not shown). To validate these observations, MIC testing was performed, which revealed that *relQ* deletion decreased oxacillin MIC 16-fold while *relP* deletion increased it 4-fold as compared to JE2. Interestingly, Δ*relPQ* was only 4-fold more sensitive than JE2 but 4-fold more resistant than the Δ*relQ* strain (Table 3). These results indicate an important role of *relQ* in β-lactam resistance expressed by MRSA, and also that deletion of *relP* and *relQ* affect the β-lactam resistance in an apparently opposite manner.

**Table 3:** Effect of *relP* and *relQ* deletion on oxacillin MIC (determined by agar double-dilution method).

**Strains/mutants**	**Oxacillin MICs (μg/ml)**	
	**(–) Mupirocin**	**(+) Mupirocin (0.03 μg/ml)**
JE2	128	256
*ΔrelP*	512	512
*ΔrelQ*	8	256
*ΔrelPQ*	32	256

### Mupirocin Restores the β-Lactam Resistance in *ΔrelQ* and *ΔrelPQ*

Since *relQ* is an active (p)ppGpp synthetase (Geiger et al., 2014), and since a higher level of this alarmone is required for the MRSA phenotype (Kim et al., 2013; Mwangi et al., 2013), it was speculated that (i) the sensitivity of Δ*relQ* might be a consequence of a *relQ* deletion-mediated decrease in (p)ppGpp level and, if so, the requirement of *relQ* for expression of β-lactam resistance should be bypassed by induction of (p)ppGpp synthesis in Δ*relQ*; and (ii) higher resistance of Δ*relPQ* than Δ*relQ* might be a consequence of (p)ppGpp synthesis by the RSH in the absence of RelP and RelQ and, if so, Δ*relPQ* resistance level should further increase by induction of (p)ppGpp synthesis via RSH. To test this, oxacillin MICs were determined in the presence of mupirocin, an isoleucine homolog capable of inducing the (p)ppGpp synthesis in *Staphylococcus* via RSH by inhibiting isoleucyl-tRNA synthetase (Cassels et al., 1995). The results indicated that mupirocin restores the β-lactam resistance in Δ*relQ* and Δ*relPQ* to the parental level (Table 3). These results suggest that *relQ* plays an important role in maintaining a higher level of (p)ppGpp required for the expression of β-lactam resistance in MRSA.

### Deletion of *relQ* Reduces the *mecA* Expression Level

**Figure 2:**
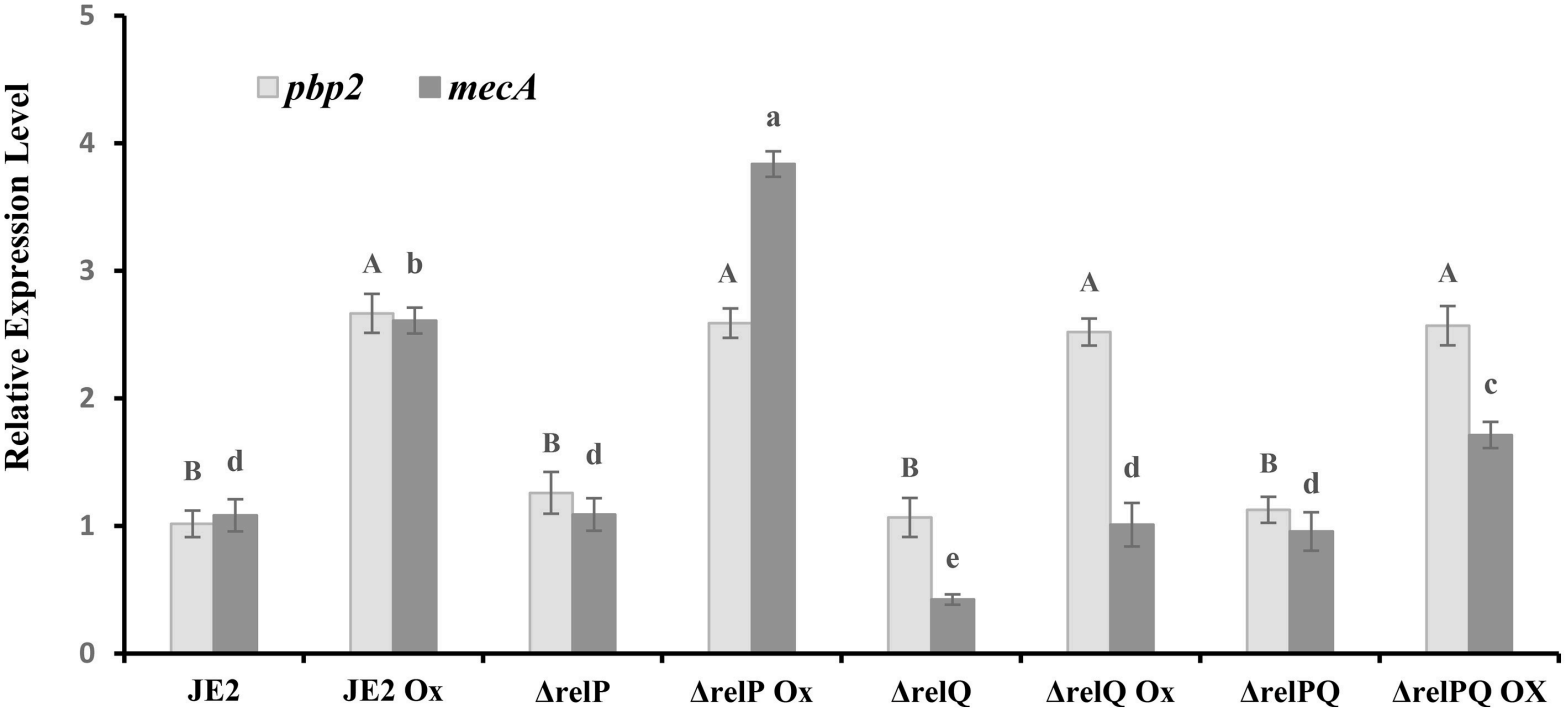
Expression analysis of *mecA* in JE2 and mutant strains. RNA was isolated from the exponentially grown (0.5–1.2 OD_600nm_) cultures treated with or without oxicillin (Ox) 4 μg/ml for 60 min in MHB. cDNA was prepared from RNA samples and used as template with appropriate primer sets, and relative expression levels were calculated as mentioned in the Materials and Methods section. Each bar shows the mean and standard deviation of values obtained from three replicates. The effect of mutation and oxacillin on expression of *pbp2* and *mecA* was analyzed by performing multiple pairwise comparisons for *pbp2* (uppercase letters) and *mecA* (lowercase letters) using ANOVA followed by Tukey's *post-hoc* test, and *p*-values < 0.05 were considered as significantly different. Different letters show statistically significant differences.

The β-lactam sensitivity of Δ*relQ* prompted us to investigate whether lack of *relQ* affected the *mecA* expression level. To examine this, relative quantification of *mecA* transcript was performed to compare its level in parent and mutants grown in the presence or absence of oxacillin. The *pbp2*, which is known to induce in response to oxacillin (Boyle-Vavra et al., 2003), was used as a positive control for this analysis and was found to follow the reported oxacillin-inducible pattern in every strain (Figure 2). Transcript analysis revealed that in JE2, Δ*relP*, and Δ*relPQ* have almost equal basal levels of *mecA* transcript; however, it is >2-fold repressed in Δ*relQ*. It is evident from Figure 2 that although oxacillin induces the *mecA* in all the strains, its level was the lowest in Δ*relQ*. It was also noted that *relP* deletion does not affect the basal level of *mecA* expression (Figure 2), but it positively affects *mecA* inducibility by oxacillin (~4-fold), providing an explanation for higher levels of β-lactam resistance in Δ*relP* than the parent. Interestingly, deletion of both the SASs restored the *mecA* basal level in Δ*relPQ* almost equal to the parent, but the oxacillin-induced level was lower than that of the parent (Figure 2). Comparison between Δ*relQ* and Δ*relPQ* revealed that the oxacillin-induced *mecA* level in Δ*relPQ* is almost 2-fold higher than that of Δ*relQ*. These results explain why the double mutant is more resistant than the Δ*relQ* mutant.

### Expression of *relP* or *relQ* Complements *ΔrelQ* and *ΔrelPQ* Mutants

**Figure 3:**
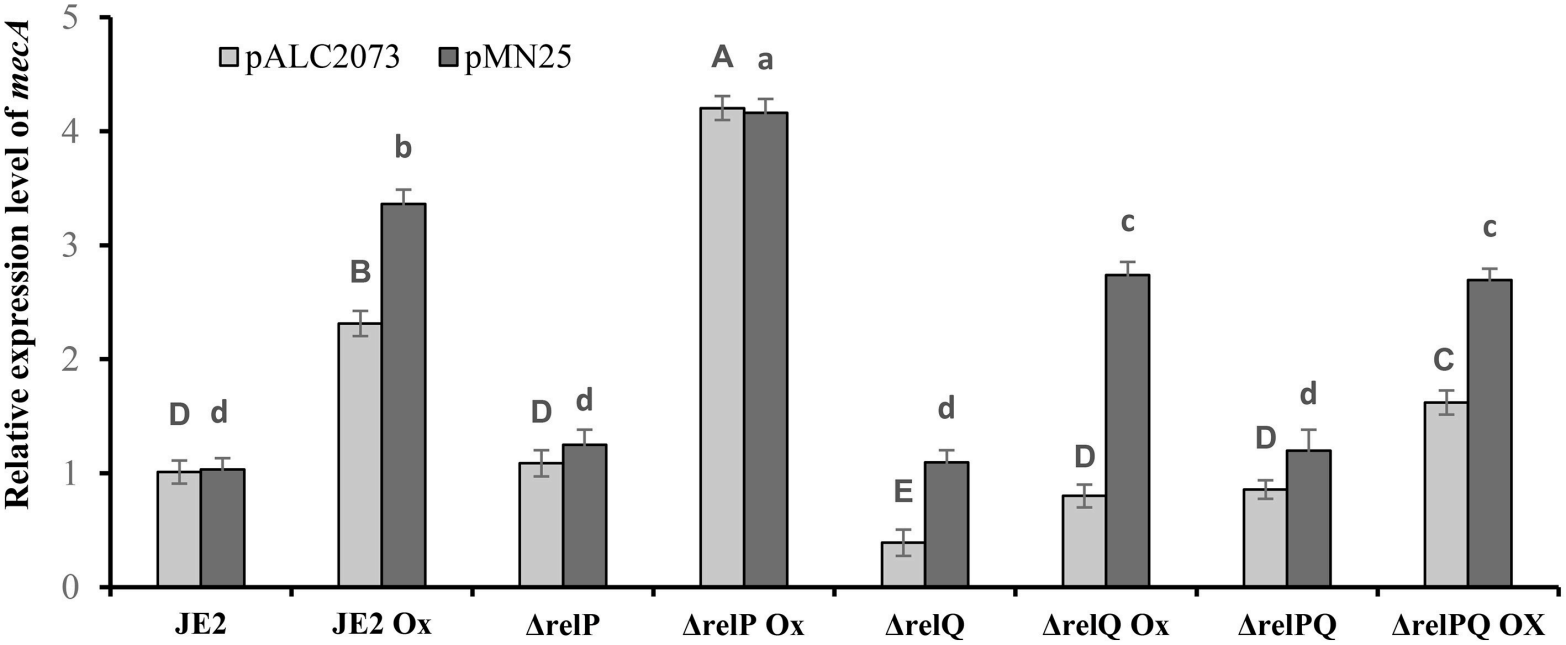
Effect of *relQ* expression on *mecA* transcription in parent and mutant strains. Transcript level was monitored by RT-PCR. cDNA was prepared by reverse-transcription of the RNA samples isolated from exponentially grown cultures treated with or without oxicillin (Ox) 4 μg/ml for 60 min in MHB. The relative expression levels were calculated using the $2^{-\text{Δ}\Delta {\text{C}}_{\text{T}}}$ method. Each bar shows the mean and standard deviation of values obtained from three replicates. The effect of *relQ* expression on *mecA* transcription level (lowercase letters) in different strains was analyzed by performing multiple pairwise comparisons using ANOVA followed by Tukey's *post-hoc* test, and *p*-values < 0.05 were considered to represent significant difference. Tukey's *post-hoc* test was also performed to analyze the effect of empty plasmid on the relative transcription level of *mecA* (uppercase letters) in different strains. Different letters show statistically significant differences.

Since both *relP* and *relQ* synthesize the same alarmone (Geiger et al., 2014), we examined whether expression of either of these can complement Δ*relQ* and Δ*relPQ*. For this, *relP* and *relQ* were cloned with their native promoters in pLI50 to construct pMN12 and pMN13, respectively (Table 1). When these plasmids were mobilized in RN4220, transformants were found for pMN12 while no transformants could be obtained for pMN13, even after several electroporations and longer incubations. We anticipated that either plasmid-borne over-expression of *relQ* or SAUSA300_0906 ORF, which encodes a hypothetical protein and is located between the promoter and *relQ* ORF (**Figure 5B**), might be a reason for such toxicity. To examine further, *relP* promoter was fused with *relQ* ORF (pMN14) and *relQ* promoter (without SAUSA300_0906 ORF) with *relP* ORF (pMN15). Transformants were obtained for pMN14 (pLI50-P_*relP*_-*relQ*), while none could be obtained for pMN15 (pLI50-P_*relQ*_-*relP*), suggesting that *relQ* promoter-driven plasmid-borne over-expression of either of the SASs is toxic to the cells. MIC determination revealed that *relP* promoter-driven plasmid-borne expression of *relP* (via pMN12) or *relQ* (via pMN14) restored resistance in Δ*relQ* and Δ*relPQ* (Table 4). To validate the *relQ* ORF-mediated complementation, pMN25 was constructed by cloning the *relQ* ORF with its native RBS into pLAC2073 to express this gene in a tetracycline-inducible manner. The MIC test revealed that pMN25-borne *relQ* expression restored the resistance in Δ*relQ* and Δ*relPQ* (Table 4). For further validation, it was examined whether *relQ* expression could restore the *mecA* expression level in the Δ*relQ* and Δ*relPQ* strains. To examine this, *mecA* transcript level was compared in parent and mutants harboring pALC2073 or pMN25 and was grown in the presence or absence of oxacillin. Figure 3 clearly shows that the expression of *relQ* restores the *mecA* level in the Δ*relQ* and Δ*relPQ* strains almost equal to the parent. Complementation of Δ*relQ* and Δ*relPQ* by plasmid-borne expression of *relP* or *relQ* validated their involvement in the expression of β-lactam resistance and ruled out the possibility of secondary mutations or polar effects in Δ*relQ* and Δ*relPQ* strains.

**Table 4:** Effect of expression of *relP, relQ*, and *mecA* on oxacillin MIC (determined by agar double-dilution method).

**Plasmids present in *S. aureus* strains/mutants**	**Oxacillin MICs (μg/ml) of** ***S. aureus*** **strains**			
	**JE2**	***ΔrelP***	***ΔrelQ***	***ΔrelPQ***
**pLI50** (Empty plasmid)	128	512	8	32
**pMN12** (pLI50-P*_*relP*_*-*relP*)	256	512	128	256
**pMN14** (pLI50-P*_*relP*_*-*relQ*)	256	512	128	256
**pALC2073** (Empty plasmid)	128	512	8	32
**pMN25** (pALC2073-*relQ*)	256	512	128	128
**pMN26** (pALC2073-*mecA*)	512	512	256	256

### Expression of *mecA* Restores β-Lactam Resistance in *ΔrelQ* and *ΔrelPQ*

The *relQ* deletion-mediated oxacillin sensitivity and reduced expression/induction of *mecA* prompted us to examine whether plasmid-borne expression by a known promoter, which is free from SR-mediated regulation, could restore oxacillin resistance in Δ*relQ* and Δ*relPQ*. For this, *mecA* ORF with its RBS was cloned into pALC2073. Comparison of MICs in the presence of 0.2 μg/ml tetracycline revealed that the Δ*relQ* and Δ*relPQ* harboring pMN26 (pALC2073-*relQ*) became oxacillin-resistant (MIC 256 μg/ml) while the presence of pALC2073 did not make any difference (Table 4). This confirmed that *relQ* deletion affects oxacillin sensitivity mainly by negatively affecting the *mecA* expression.

### *relQ* Promoter Is Stronger and Responds to Lack of *relP* and *relQ*

**Figure 4:**
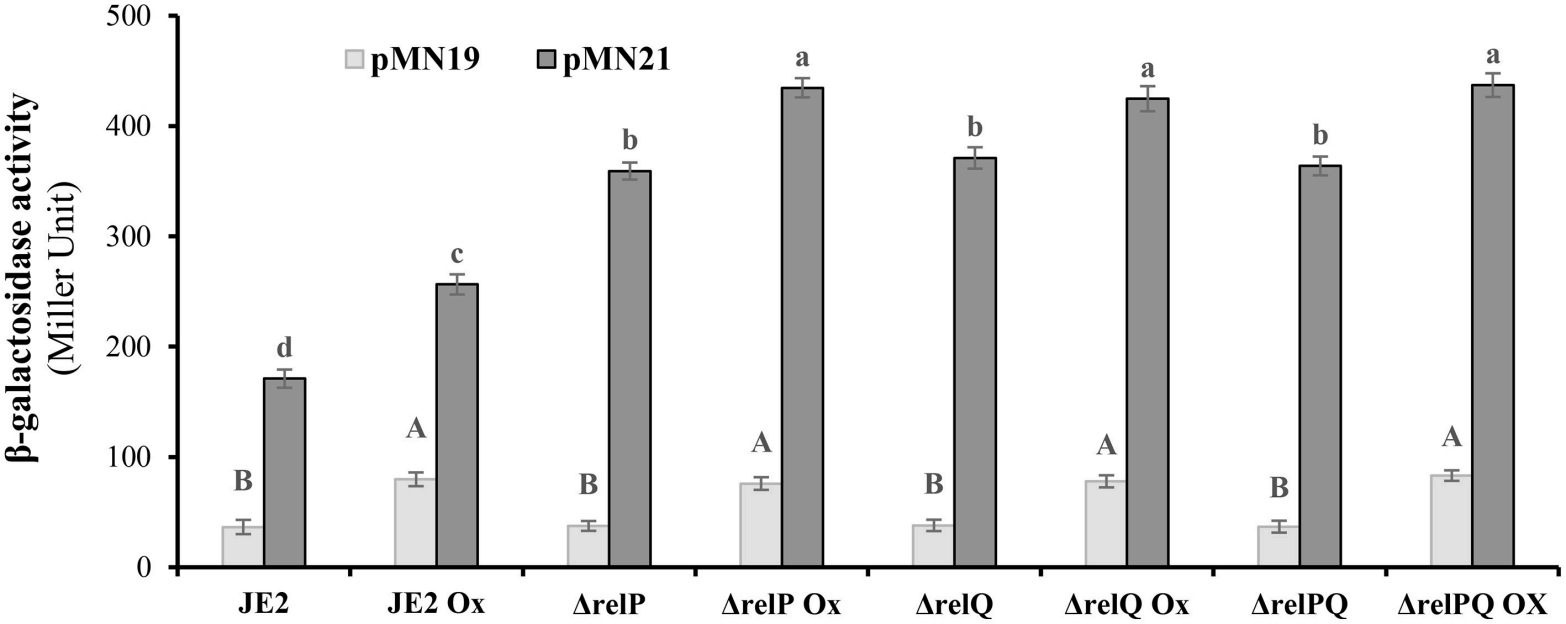
β-galactosidase activity of JE2 and its mutants harboring *lacZ* transcriptionally fused to *relP* (pMN19) and *relQ* (pMN21) promoter. β-galactosidase activity was performed in triplicate at three different occasions with cells harvested from exponentially grown (0.6–1.2 OD_600nm_) cultures treated with or without oxicillin (Ox) 4 μg/ml for 60 min in MHB. Data for empty plasmid (pMN18) are omitted because of its undetectable β-galactosidase activity. Data for pMN20 (SAUSA300_0906 promoter:*lacZ* fusion) are not included, as it gives the activity equal to the pMN21. Each bar shows a mean and standard deviation of values obtained from three replicates. The effect of mutation and oxacillin on the promoter activity of *relP* and *relQ* was analyzed by performing multiple pairwise comparisons of β-galactosidase activities in different strains due to *relP* (uppercase letters) or *relQ* (lowercase letters) promoter using ANOVA followed by Tukey's *post-hoc* test, and *p*-values < 0.05 were considered to represent significant difference. Different letters show statistically significant differences.

Since an induced level of this alarmone is required for β-lactam resistance (Mwangi et al., 2013), it was hypothesized that positive effects of *relP* deletion on β-lactam resistance might be a consequence of *relQ* induction in the *relP-*deleted background. To examine this, *relP/Q* promoter activities were monitored in response to their deletions and oxacillin. For this, *E. coli lacZ* ORF was engineered and cloned with a terminator into the XbaI-HinDIII site of pLI50 to construct reporter vector pMN18 (as described in the Materials and Methods section). Using pMN18, the upstream regions of *relP* (485 bp), SAUSA300_0906 (231 bp), and *relQ* (599 bp, including SAUSA300_0906 ORF and the 231 bp upstream region), without their RBS, were transcriptionally fused with *lacZ* to construct pMN19, pMN20, and pMN21, respectively. These constructs were mobilized into parent and mutant strains via *S. aureus* RN4220. Isolation of transformant for pMN21 (SAUSA300_0906 with its upstream (*relQ*) promoter region) indicated that plasmid-borne over-expression of SAUSA300_0906 was not toxic to the cells; this confirmed that *relQ* promoter-driven plasmid-borne over-expression of the *relQ* was the reason for the toxicity observed during transformation of pMN13. β-galactosidase assays revealed that the empty vector (pMN18) resulted in zero activity, and pMN20 and pMN21 resulted in equal activity (data not shown), suggesting that *relQ* is transcribed from the promoter located upstream of SAUSA300_0906. Results showed that, (i) *relP* promoter activity was equal in all the strains, and oxacillin induces it ~2-fold in every strain; (ii) *relQ* promoter is ~5-fold stronger than *relP* and induced (~1.5-fold) by oxacillin; and interestingly (iii) its activity is 2-fold higher in *relP*- and/or *relQ*- deleted backgrounds compared to that of the parent (Figure 4). These results suggest that the *relP* promoter responds only to oxacillin while the *relQ* promoter induces in response to oxacillin as well as the lack of *relP* and/or *relQ*, which enables it to express *relQ* efficiently in the Δ*relP* strain.

### Identification of *relP* and *relQ* TSSs

**Figure 5:**
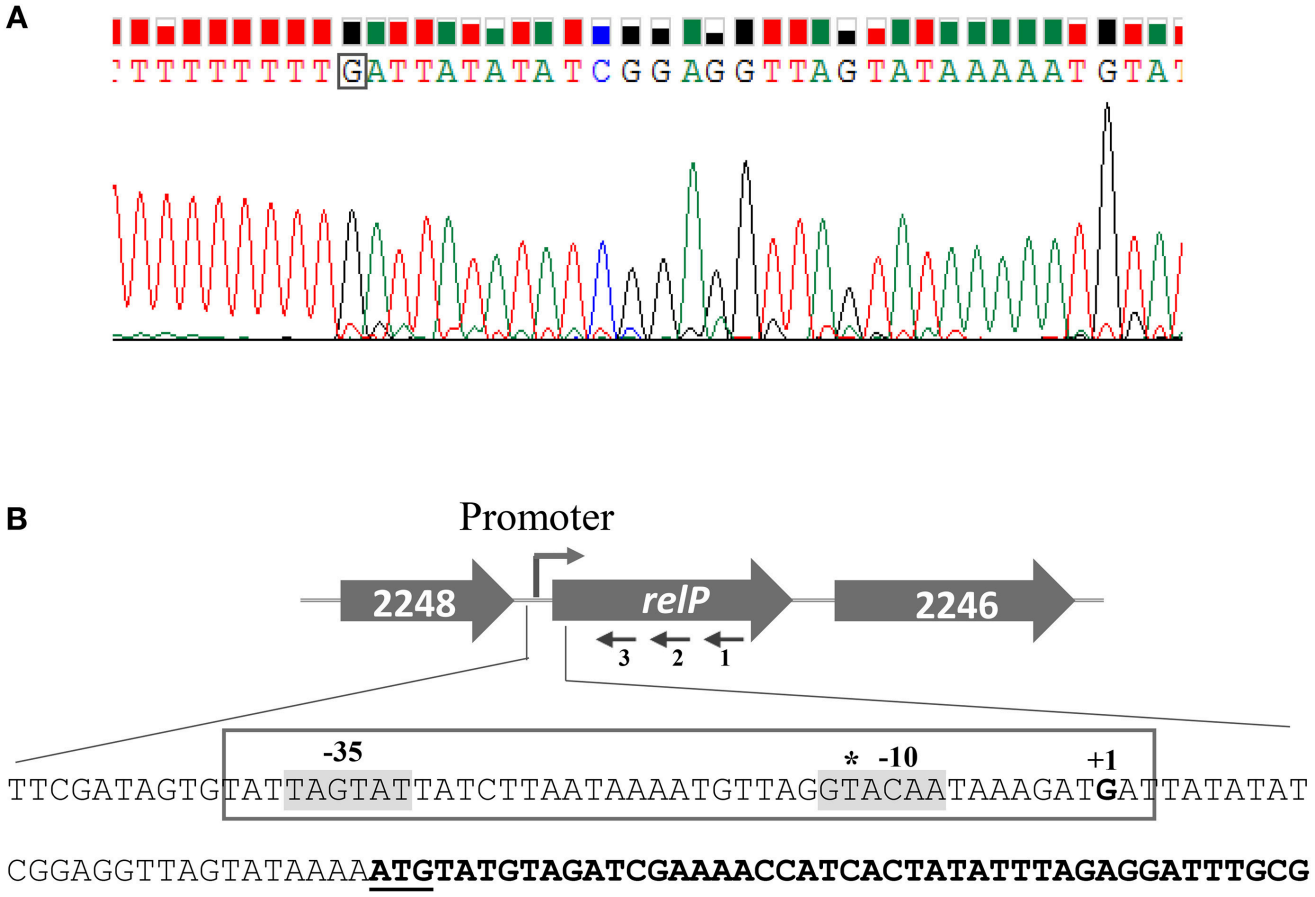
Determination of the *relP* transcription start site (TSS) by 5′ RACE. **(A)** Electropherogram, showing TSS (boxed nucleotide), is representative of results from sequencing of several distinct clones obtained after 5′ RACE experiments. **(B)** Schematic representation of *S. aureus relP* chromosomal region. Large and filled arrows represent the relative size, location, and transcriptional orientation of ORFs. Small and thin arrows represent the regions used to design primers for 5′RACE experiment; relP-SP1:R (1), relP-SP2:R (2), and relP-SP3:R (3). Nucleotide sequences from −78 to +44 of *relP* start codon (underlined) showing TSS (indicated as +1) and possible −35 and −10 elements (gray background). *relP* ORF is indicated by bold nucleotides. The earlier predicted TSS is shown by asterisk.

**Figure 6:**
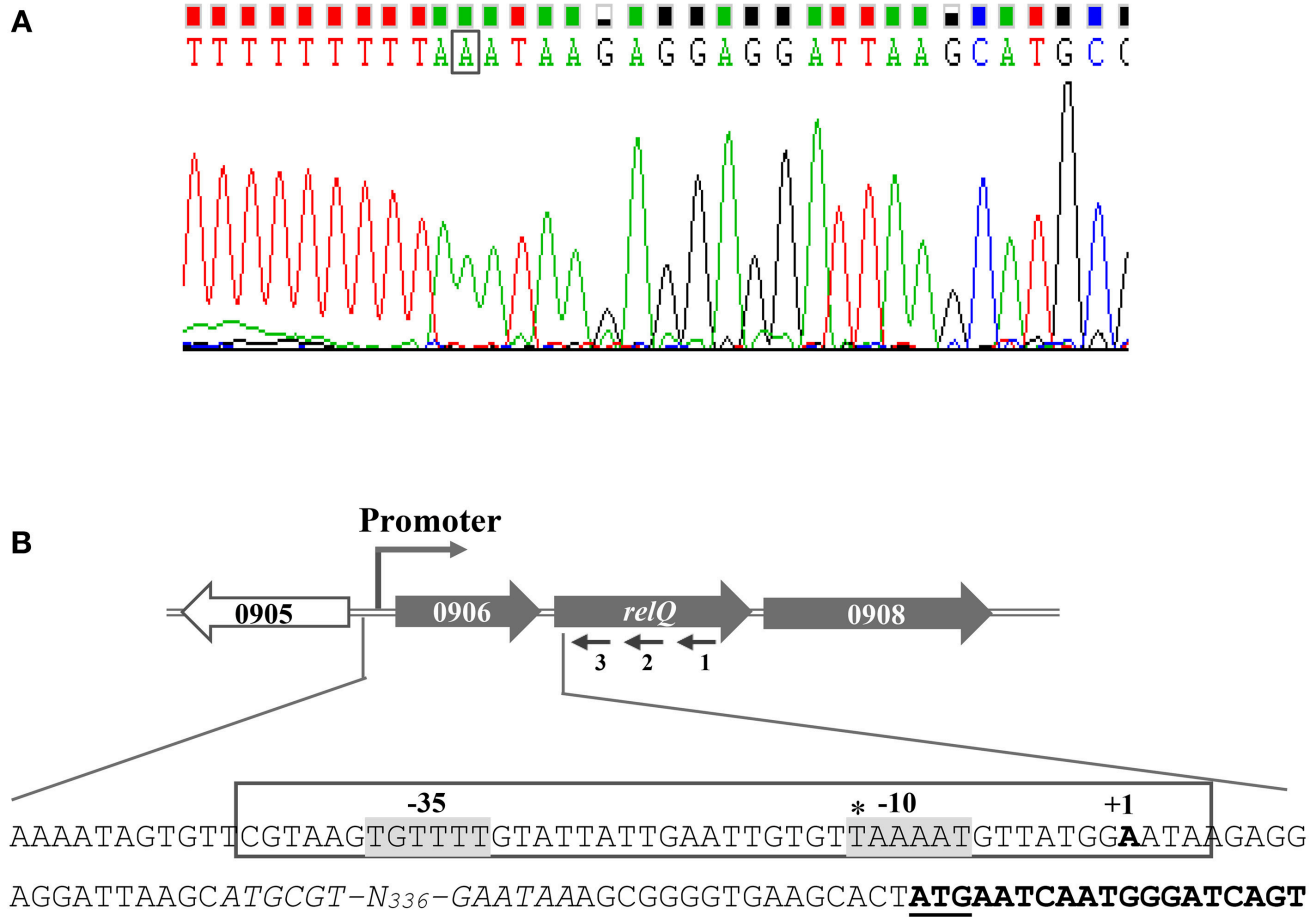
Determination of the transcription start site (TSS) of *relQ* by 5′ RACE. **(A)** Electropherogram showing TSS (boxed nucleotide) is representative of results from sequencing of several distinct clones obtained after 5′ RACE experiment. **(B)** Schematic representation of *S. aureus relQ* chromosomal region. Large and filled arrows represent the relative size, location, and transcriptional orientation of ORFs. Small and thin arrows represent the regions used to design primers for the 5′RACE experiment; relQ-SP1:R (1), relQ-SP2:R (2), and relQ-SP3:R (3). Nucleotide sequences from −436 to +19 of *relQ* start codon (underlined) showing TSS (indicated as +1) and possible −35 and −10 elements (gray background). SAUSA300_0906 ORFs are shown by italicized nucleotides, and the portion of *relQ* ORF is shown in bold. The earlier predicted TSS is shown with an asterisk.

Difference in the promoter activity/inducibility of *relP/Q* prompted us to examine the differences in their promoter elements. Although *relP/Q* TSSs have been mapped and primary sigma factor (SigA) binding motifs have been predicted in their upstream regions using transcriptomic approaches (Mader et al., 2016), the mapped TSSs have been shown to have an upshift tendency, which creates problems in identification of actual TSSs and promoter motifs. 5′ RACE was performed to identify the actual TSSs, which allowed the identification of their possible −35 and −10 elements. The identified TSSs are 27 and 19 bp upstream of *relP* and SAUSA300_0906 start codons, respectively (Figures 5, 6). These are 13 and 14 bp downstream of the earlier reported TSSs of *relP* and *relQ*, respectively. Sequence analysis revealed that *relP* had TAGTAT (−35) and GTACAA (−10), and *relQ* had TGTTTT (−35) and TAAAAT (−10) promoter elements. This indicates a significant difference in their −35 and −10 elements that might be the reason for difference in their promoter activities.

## Discussion

Several studies have shown that SR, which is established by an increased level of (p)ppGpp, is involved in bacterial stress adaptation, drug resistance, pathogenesis and persistence. Recently, involvement of (p)ppGpp in the expression of β-lactam resistance was reported by showing that laboratory or clinical hetero-resistant MRSA strains required an induced level of (p)ppGpp (either by exposure to mupirocin or truncation of RSH) for the expression of homogeneous and increased levels of oxacillin resistance (Kim et al., 2013; Mwangi et al., 2013). Because every MRSA isolate does not carry a truncated RSH or need exposure of mupirocin-like SR-inducers, how (p)ppGpp synthesis is induced in response to β-lactams in MRSA is still not clear. Since exposure of β-lactams is not supposed to pose any nutrient starvation, direct involvement of RSH in β-lactam-induced (p)ppGpp synthesis does not seem logical. With these considerations, we initiated this study based on a hypothesis that RelP/Q might be involved in (p)ppGpp synthesis in response to β-lactams because these SASs are active (p)ppGpp synthetases and induced in response to cell-wall stresses in *S. aureus* (Geiger et al., 2014).

Although recent reports show that RelP/Q are induced in response to cell-wall stresses to mitigate such conditions in MSSA (Geiger et al., 2014), their contribution to the expression of β-lactam resistance in MRSA is still unclear. To examine their roles, we deleted *relP* and *relQ* in a CA-MRSA and kept RSH intact to emphasize their function. Since the (p)ppGpp synthetic activity of these SASs has been reported *in vivo* and *in vitro* (Geiger et al., 2014), we did not feel the need to measure the (p)ppGpp level in the SAS-deleted strains, though lack of (p)ppGpp estimation facility was another reason. Our observation that deletion of *relQ* renders CA-MRSA sensitive to several β-lactams suggests an important role of RelQ in the expression of β-lactam resistance. This finding corroborates an earlier observation that a laboratory-generated highly oxacillin-resistant *S. aureus* strain reverted to a reduced-resistance strain due to a point mutation in the synthetase domain of RelQ (Mwangi et al., 2013). Since RelQ synthesizes (p)ppGpp, it was examined whether mupirocin-induced (p)ppGpp synthesis could bypass the requirement of RelQ for β-lactam resistance. The results showed that mupirocin fully restored the resistance in Δ*relQ*, suggesting that *relQ* deletion increases the sensitivity mainly by negatively affecting the (p)ppGpp level.

Our observations that *relQ* deletion reduces the basal level expression and oxacillin inducibility of *mecA* provides an explanation for β-lactam sensitivity of Δ*relQ*. Complementation of Δ*relQ* by a *xyl/tetO* promoter-driven *mecA* expression further validates that *relQ* deletion increases sensitivity by reducing *mecA* expression. Interestingly, we observed that deletion of both the SASs renders Δ*relPQ* more resistant than Δ*relQ* by restoring the *mecA* expression level almost equal to the parent. Since requirement of (p)ppGpp for *mecA* expression has been shown (Kim et al., 2013; Mwangi et al., 2013), it seems logical to hypothesize that the reduced sensitivity of Δ*relPQ* might be a consequence of RSH-mediated synthesis of (p)ppGpp to maintain the basal level of this alarmone in the absence of both the SASs. Our data also show that although the basal level of *mecA* is restored in Δ*relPQ*, its oxacillin-induced level was lower than the parent, indicating the importance of RelP/Q for efficient induction of *mecA* in response to β-lactams.

Lack of polar effects or secondary mutations is supported by our genetic complementation data, which shows that *xyl/tetO* promoter-driven expression of *relQ* fully complements the mutant. It was also observed that multi-copy plasmid-borne *relP* promoter-driven expression of *relP* or *relQ* ORF complements the mutants while *relQ* promoter-driven plasmid-borne expression of either of these results in lethality. It appears that *relP* promoter activity is insufficient for the expression of *relP* to compensate the effect of *relQ* deletion when it is present as a single copy on the Δ*relQ* chromosome, but its presence on a multi-copy plasmid amplified the promoter activity and complemented the mutant. Based on promoter activity data, which showed that *relQ* promoter is >5-fold stronger than the *relP*, it can be inferred that the toxicity associated with *relQ* promoter-driven plasmid-borne expression of either of the SASs might be a consequence of a high level of (p)ppGpp due to further amplification of the *relQ* promoter activity by the plasmid copy-number. However, *relQ* promoter-driven expression of *relQ* ORF from a single chromosomal copy is not toxic to *S. aureus*.

In addition to revealing the importance of *relQ*, the results also indicate a positive effect of *relP* deletion on β-lactam resistance. Expression analysis provides an explanation by showing that *relP* deletion increases the oxacillin inducibility of *mecA*. Our data shows that *relQ* promoter is induced in response to oxacillin as well as deletion of either of the SASs while the *relP* promoter responds only to oxacillin. These observations suggest that *relQ* is efficiently expressed in the *relP*-deleted background, which enhances the *mecA* expression and renders the Δ*relP* strain highly resistant. Inducibility of *relQ* promoter in *relP*- and/or *relQ*-deleted backgrounds indicates toward its ability to induce in response to low (p)ppGpp levels. Although SigA binding motifs have been predicted in *relP/Q* upstream regions, their −35 and −10 elements have not been identified because the mapped TSSs have an upshift tendency (Mader et al., 2016). By promoter mapping, we have identified the TSSs and the most probable −35 and −10 elements. The results revealed that the identified −35 elements of *relP* (TAGTAT) and *relQ* (TGTTTT) showed similarity with the reported SigA −35 (TGATAA and TTTATT) consensus elements (Deora and Misra, 1996), which supports their SigA-dependence predicted earlier (Geiger et al., 2014; Mader et al., 2016). However, differences in their −35 and −10 elements provide an insight into difference in their promoter activities.

Recently, it was shown that although RelQ deletion does not affect the level of β-lactam resistance in N315 and Mu3, its overexpression increases the resistance level several-fold (Matsuo et al., 2019). These studies suggest that RelQ plays an important role in the expression of β-lactam resistance, but the requirement of its expression level may depend on the strains. Based on our findings, we propose that possibly a certain level of (p)ppGpp is required for the expression of *mecA* in JE2, and both the SASs are involved in maintaining this level, which might be affected by deletion of either of these. However, an enhanced oxacillin-inducibility of *relQ* in the Δ*relP* compensates for the effect of *relP* deletion, while the effect of *relQ* deletion cannot be compensated due to the lack of *relP* inducibility in response to *relQ* deletion. This difference in regulation of *relP* and *relQ* results in sensitivity in Δ*relQ* and resistance in Δ*relP*. Our findings show that RelQ mediates the expression of *mecA* in response to β-lactams in MRSA, but how these SASs are regulated differently is a matter of further research.

## Author Contributions

AB performed most of the experiments and compiled the data. PP, AD, and AZ helped in promoter mapping and promoter activity analysis. GN helped in data analysis and discussion. MM designed the study and experiments, interpreted the results, and wrote the manuscript.

### Conflict of Interest Statement

The authors declare that the research was conducted in the absence of any commercial or financial relationships that could be construed as a potential conflict of interest.
