# Supplementary material for: RelQ Mediates the Expression of β-Lactam Resistance in Methicillin-Resistant Staphylococcus aureus
Source: Front Microbiol. 2019 Mar 11;10:339. doi: 10.3389/fmicb.2019.00339 (PMC6421274; doi:10.3389/fmicb.2019.00339)
Supplement: Supplementary file 1 [file Data_Sheet_1.docx]

**Supplementary Figure 1.** Agarose-gel showing the amplicons obtained with relP-up:F/relP:R and relQ-up:F/relQ:R primer sets using genomic DNA from JE2 (Lane 1), *ΔrelP* (Lane 2 &3), *ΔrelQ* (Lane 4 & 5) and *ΔrelPQ* (Lane 6 & 7) strains as a template. relP-up:F /relP:R primer set is supposed to produce 1209 bp amplicon with JE2 or *ΔrelQ* genomic DNA and a smaller amplicon of 532 bp with that of *ΔrelP* or *ΔrelPQ*. Similarly, the relQ-up:F/relQ:R primer set is supposed to produce 1616 bp amplicon with JE2 or *ΔrelP* genomic DNA and a 998 bp amplicon with that of *ΔrelQ* or *ΔrelPQ*. Lane 8 shows the different fragments of 1 kb plus DNA ladder (Invitrogen).


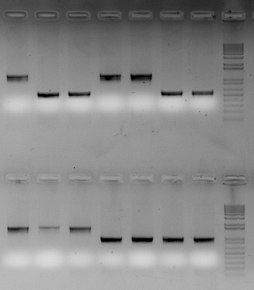


**relP-up:F /**

**relP:R**

**relQ-up:F /**

**relQ:R**

**1.65 kb**

**0.5 kb**

**1.0 kb**

**1 2 3 4 5 6 7 8**

***ΔrelP***

***ΔrelQ***

***ΔrelPQ***

**JE2**
